# Revised Subjects of the Current Korean Oriental Pharmacists' Licensing Examination

**DOI:** 10.3352/jeehp.2007.4.4

**Published:** 2007-12-20

**Authors:** Jong-Pil Lim, Seon-Pyo Hong, Young-Mi Lee, Hoon Jeon

**Affiliations:** 1 Department of Oriental Pharmacy, College of Pharmacy, Woosuk University, Wanju, Korea.; 2 Department of Oriental Pharmacy, College of Pharmacy, Kyunghee University, Seoul, Korea.; 3 Department of Oriental Pharmacy, College of Pharmacy, Wonkwang University, Iksan, Korea.

**Keywords:** Validity, Korean Oriental Pharmacists' Licensing Examination, Subjects

## Abstract

This study is designed to draw out new integrated subjects of the Korean Oriental Pharmacists' Licensing Examination (KOPLE). In 2004, for the revision of subjects, we have analyzed the curriculums of the Oriental Pharmacy department, the oriental pharmacist's (OP's) job description book, and the elementary items of KOPLE. We also examined the system of the Chinese Herb Pharmacists' Examination and other health personnel licensing examinations and studied the data of items and compared them with KOPLE. We heard the public opinion on the present KOPLE. We developed a subfield of 18 subjects, a middle category of 188 items, and a small category of 1,026 items. We proposed a new KOPLE that consists of three subjects: basic oriental pharmacy, applied oriental pharmacy, and laws and regulations.

## INTRODUCTION

This study is to draw out the new unified subjects of the Korean Oriental Pharmacists' Licensing Examination (KOPLE) by examining the items of the KOPLE, and the curriculum and job description of oriental pharmacists (OPs). This work may help OP students have a capacity of synthetic thinking, which is required in the clinical situation. Also, we tried to determine the integrated subjects of the KOPLE for a future-oriented KOPLE. Finally, we arranged it into large, middle, and small categories.

## MATERIALS AND METHODS

We analyzed the curriculums of oriental pharmacy departments [1-3], the oriental pharmacist's (OP's) job descriptions book [4], the elementary items of the KOPLE, the system of the Chinese Herb Pharmacists' Examination, and other similar health personnel licensing examinations. We also heard the opinions of oriental pharmacy professors, OPs, and would-be OPs. After that, we proposed integrated subjects for the KOPLE.

## RESULTS

**Fig. 1:**
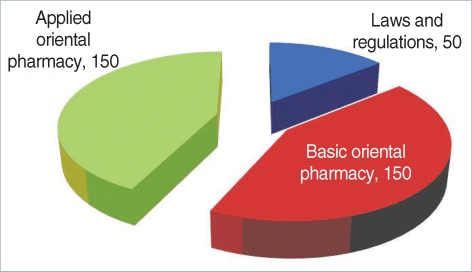
The unified KOPLE. Unit: points.

The results showed that every Oriental Pharmacy department in Korea has been offering all subjects focused on the KOPLE, obviously implying that the KOPLE has been an absolute influence on the curriculums of the Oriental Pharmacy (Table 1). According to the results of an analysis of the data of the OP's job descriptions book, there are quite a lot of differences between the importance of the book and the proportion of KOPLE items, so that the book doesn't contain an equal proportion of KOPLE items. Therefore, it can be said that the current method of the KOPLE has mainly been set on knowledge-focused items regarding the evaluation of OP capability, as many as about 68. From the analysis of the passing rates on KOPLE for the past 3 years, there is no problem on the level of examination difficulty, the discrimination rate, and the passing rate. The system of the Chinese Herb Pharmacists' Examination has shown that the number of items is 400, which is 1.6 times as many as the ones in KOPLE (250), and the linking subjects integrated into 4 subjects have been administered, which is an upgraded system over the one in Korea (Table 2). On examination, in some similar health personnel licensing examinations, the number of KOPLE items approaches 45.5% compared with 550 (538 points), which is the number of medical licensing examination items, and is 50-80 less than the items set in pharmacists', nurses', and nutritionists' licensing examinations (Table 2). Based on Bloom's theory, research of the items among oriental pharmacy professors and OPs on the validity of the current KOPLE surveyed in 5 points that measure method has been carried out. The result has brought out that the overall validity rate of KOPLE, the degree that KOPLE reflects the curriculum of the oriental pharmacy, and the view on the measurement of the lowest capability for OPs by KOPLE shows over 3 points that signify positive meaning, but the view on the measurement of the OP's problem-solving capacity shows less than 3 points, which is negative. The reason that the KOPLE should be integrated into 3 subjects is that the KOPLE is reciprocally related to one or the other subjects. A survey on the number of items in KOPLE has shown that the professors have an absolute preference for 350 items because it is necessary for the items to be increased for adequate evaluation through the KOPLE. Through a study of the data of items and a public hearing for the present KOPLE, it has been arranged that the KOPLE subjects are 'LAWS AND REGULATIONS', 'BASIC ORIENTAL PHARMACY', and 'APPLIED ORIENTAL PHARMACY'. The contents of subject 'LAWS AND REGULATIONS' are 'The Law of Pharmaceuticals and Narcotics', 'Korean Pharmacopoeia', 'Korean Herbal Pharmacopoeia' and 'The Law for Marketing of Herbal Medicine (HM)'. Contents of subject 'BASIC ORIENTAL PHARMACY' are 'Pharmaceutical Analysis', 'Pharmaceutical Botany', 'Pharmacognosy', 'Natural Products Chemistry', 'Herbalogy', 'Hygienic Chemistry' and 'Pharmaceutical Biochemistry'. The contents of subject 'APPLIED ORIENTAL PHARMACY' are 'Basics of Oriental Pharmacy', 'Processing of HM', 'Preparation of HM', 'Pharmacology of HM', 'Oriental Pharmaceutics', 'Marketing and Storing of HM' and 'Pharmacology'. Number of items of each subject is 50 for 'LAWS AND REGULATIONS' and 150 each for 'BASIC ORIENTAL PHARMACY' and 'APPLIED ORIENTAL PHARMACY' (Table3, Fig. 1). Through collecting the extensive opinions and suggestions from the concerned professors and other professors on the KOPLE, we have arranged the KOPLE into large, middle, and small categories. There are 3 large categories, and 18 subjects as the subfield, 188 items as the middle category, and 1,026 small category items (Table 4).

## DISCUSSION

This study was desgned to propose the new integrated subjects of KOPLE, Three subjects are proposed like other health personnel licensing examination. Recently, the trends of the subjects of health personnel licensing examination is the integration and the emphasis of the practical skills. In this trends, subjects of KOPLE was revised. This is the first stage of the progress for KOPLE. Furthermore, the other performance test such as practical skill test, should be considered to be applied to KOPLE.

## Figures and Tables

**Table 1:**
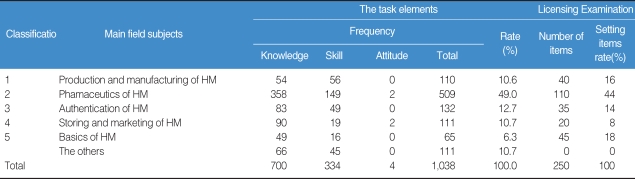
The task elements and proportion in the Korean Oriental Pharmacists' Licensing Examination listed in the oriental pharmacist's (OP's) job descriptions book

HM: Herbal Medicine.

**Table 2:**
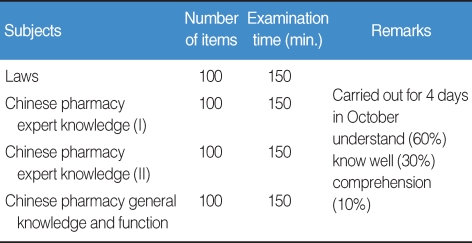
Chinese herb pharmacists' examination

**Table 3:**
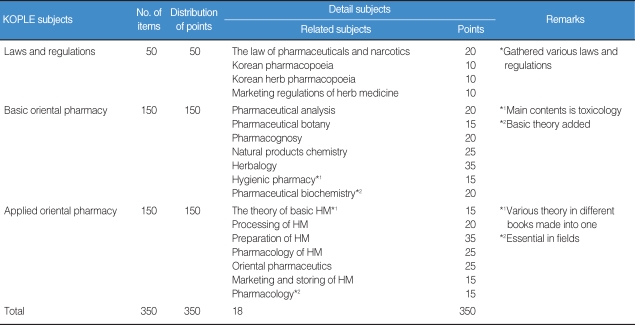
The unified KOPLE (draft)

**Table 4:**
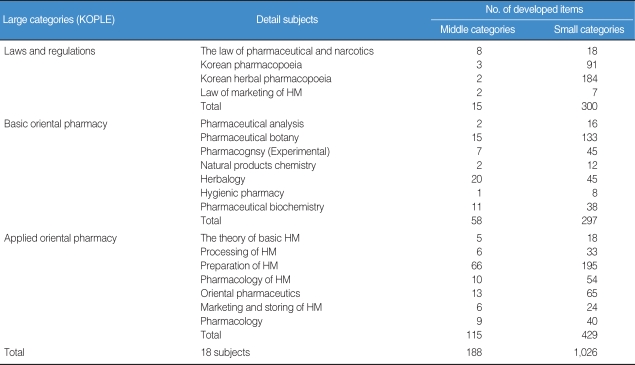
The number of developed items in the KOPLE
